# TNF-α Pretreatment Improves the Survival and Function of Transplanted Human Neural Progenitor Cells Following Hypoxic-Ischemic Brain Injury

**DOI:** 10.3390/cells9051195

**Published:** 2020-05-11

**Authors:** Miri Kim, Kwangsoo Jung, Younhee Ko, Il-Sun Kim, Kyujin Hwang, Jae-Hyung Jang, Jeong Eun Shin, Kook In Park

**Affiliations:** 1 Yonsei Biomedical Research Institute, Yonsei University College of Medicine, Seoul 03722, Korea; mrfpno@yuhs.ac (M.K.); whitesnown7@yuhs.ac (I.-S.K.); jin8140@yuhs.ac (K.H.); 2 Department of Pediatrics, Severance Children’s Hospital, Yonsei University College of Medicine, Seoul 03722, Korea; aslk82@yuhs.ac; 3 Division of Biomedical Engineering, Hankuk University of Foreign Studies, Yongin 17035, Korea; younhee.ko@gmail.com; 4 Brain Korea 21 Plus Project for Medical Science, Yonsei University College of Medicine, Seoul 03722, Korea; 5 Department of Chemical and Biomolecular Engineering, Yonsei University, Seoul 03722, Korea; j-jang@yonsei.ac.kr

**Keywords:** tumor necrosis factor-alpha, human neural progenitor cells, hypoxic-ischemic brain injury, cell survival, cellular inhibitor of apoptosis 2, CX3CL1

## Abstract

Neural progenitor cells (NPCs) therapy offers great promise in hypoxic-ischemic (HI) brain injury. However, the poor survival of implanted NPCs in the HI host environment limits their therapeutic effects. Tumor necrosis factor-alpha (TNF-α) is a pleiotropic cytokine that is induced in response to a variety of pathological processes including inflammation and immunity. On the other hand, TNF-α has protective effects on cell apoptosis and death and affects the differentiation, proliferation, and survival of neural stem/progenitor cells in the brain. The present study investigated whether TNF-α pretreatment on human NPCs (hNPCs) enhances the effectiveness of cell transplantation therapy under ischemic brain. Fetal brain tissue-derived hNPCs were pretreated with TNF-α before being used in vitro experiments or transplantation. TNF-α significantly increased expression of cIAP2, and the use of short hairpin RNA-mediated knockdown of cIAP2 demonstrated that cIAP2 protected hNPCs against HI-induced cytotoxicity. In addition, pretreatment of hNPCs with TNF-α mediated neuroprotection by altering microglia polarization via increased expression of CX3CL1 and by enhancing expression of neurotrophic factors. Furthermore, transplantation of TNF-α-treated hNPCs reduced infarct volume and improved neurological functions in comparison with non-pretreated hNPCs or vehicle. These findings show that TNF-α pretreatment, which protects hNPCs from HI-injured brain-induced apoptosis and increases neuroprotection, is a simple and safe approach to improve the survival of transplanted hNPCs and the therapeutic efficacy of hNPCs in HI brain injury.

## 1. Introduction

Neonatal hypoxic-ischemic (HI) brain injury is a major cause of neonatal mortality and severe neurological disability, including mental retardation, epilepsy, learning disabilities, and cerebral palsy [1,2]. Currently, many experimental therapies have shown limited success in the treatment of perinatal asphyxia in the clinical environment [3]. Among the many approaches to improve neurological function and reduce infarction, stem cell transplantation has shown considerable promise. For example, transplantation of self-renewing neural progenitor cells (NPCs) with the ability to differentiate into neurons and glial cells induces tissue regeneration and restores neurological function after ischemic brain injury [4,5,6]. However, the benefits of NPC therapy are limited by the poor survival, integration, differentiation, and functional neural connection in the inhospitable environment of the injured central nervous system (CNS) [6,7,8,9,10,11]. Therefore, strategies such as genetically modifying stem cells to overexpress pro-survival signaling-related or paracrine factors have been suggested to enhance the survival of transplanted cells and the effectiveness of cell transplant therapy [12,13,14,15]. However, although genetic modification of stem/progenitor cells has improved transplant outcomes, a simpler and safer approach may be needed for future clinical application of stem cell therapies. Pretreatment of NPCs with pharmacological agents might result in significant cytoprotective effects and improve functional outcomes against CNS disorders [16,17,18].

Tumor necrosis factor-alpha (TNF-α) is a pleiotropic cytokine that is induced in response to a variety of pathological and physiological processes including inflammation and immunity. TNF-α is present at high levels in various neurodegenerative disorders such as Parkinson’s disease, Alzheimer’s disease, Huntington’s disease, amyotrophic lateral sclerosis, and stroke, where it is thought to play an important role as a proinflammatory agent [19,20]. In recent years, several studies have reported that TNF-α has protective effects on cell apoptosis and death including endothelial cells and neurons, with it affecting the differentiation, proliferation, and survival of neural stem/progenitor cells in the brain, resulting in tissue regeneration and neurological function recovery after stroke, neurodegeneration, and inflammation [21,22,23]. In accordance with these results, our previous study showed that TNF-α pretreatment plays an important role in the protection of human NPCs (hNPCs) against oxygen-glucose deprivation-induced apoptosis [24]. However, further investigation is needed to determine whether it can increase the survival of transplanted hNPCs and improve neurological function in HI brain injury.

In the present study, we applied a preconditioning protocol to treat hNPCs with TNF-α prior to transplantation into neonatal mice with HI brain injury. We then observed the survival rate of engrafted hNPCs and the therapeutic effect of TNF-α-preconditioned hNPCs on brain function in HI brain injury.

## 2. Materials and Methods

### 2.1. Culture of hNPCs

Human fetal brain tissue derived from a cadaver at 13 weeks of gestation was obtained with full parental consent and approval of the Research Ethics Committee of Yonsei University College of Medicine, Seoul, Korea (Permit Number: 4-2003-0078 the date of approval is December 19,2003 ) [25]. All procedures conformed to the National Institutes of Health and Korean Government guidelines. hNPCs isolated from the telencephalon were grown as neurospheres in Dulbecco’s modified Eagle’s medium and Ham’s F-12 medium (DMEM/F12; Gibco, Carlsbad, CA, USA) containing 1% N2 formulation (Gibco), 1% penicillin/streptomycin (P/S; Gibco), 10 ng/mL leukemia inhibitory factor (LIF; Sigma, St. Louis, MO), 20 ng/mL fibroblast growth factor-2 (FGF2; R&D Systems, Minneapolis, MN, USA), and 8 μg/mL heparin (Sigma). All cultures were maintained at 37 °C in a humidified incubator containing 5% CO_2_ and air. Half of the growth medium was replenished every 3–4 d. For TNF-α (Peprotech, Rocky Hill, NJ, USA) pretreatment, TNF-α was added to the cell culture medium with a final concentration of 20 ng/mL for 24 h and then washed out before in vitro experiments or transplantation.

### 2.2. HI Brain Injury Model and Cell Transplantation

All procedures were approved by the Institutional Animal Care and Use Committee at Yonsei University College of Medicine, Seoul, Korea (Permit Number: 2015-0378 the date of approval is February 2, 2016). At 7 days after birth, ICR mice were subjected to permanent right common carotid artery occlusion under isoflurane anesthesia. Sham-operated animals were anesthetized, and a midline neck incision was made, but the carotid artery was not ligated. After recovery for 1 h, except of the sham group, the vehicle control and experimental animals were exposed to hypoxia with 8% oxygen and 92% nitrogen for 1.5 h. Body temperature was maintained at 37 °C using a warm pad. All mice received the same care and housing, and were evenly distributed between control and treatment groups. On day three after induction of HI brain injury, the mice were divided randomly and equally into three groups for hNPC or vehicle injection: a vehicle group, an hNPC group, and a TNF-α-pretreated hNPC group. The mice were anesthetized, dissected through the dorsal midline of the scalp, and injected with TNF-α-pretreated hNPCs, non-pretreated hNPCs (10 μL of cell suspension at 8 × 10^4^ cells/μL), or the same amount of vehicle (Hanks’s balanced salt solution containing 10 mM HEPES; H-H buffer; Gibco) into the center of the infarcted region using a glass micropipette (0.3 mm diameter). Once a day, cyclosporine (10 mg/kg; Sandimmune, Novartis Korea, Seoul) was administered intraperitoneally to all groups of mice from the day before transplantation until sacrifice.

### 2.3. Transfection with cIAP2-Targeting Short Hairpin RNA (shRNA)

A self-inactivating lentivirus was prepared by transient transfection of 293T cells as described previously [24]. Briefly, 20 μg of the lentiviral plasmid pLKO-shcIAP2-A (a gift from William Hahn; Addgene, Cambridge, MA, USA) or the control scrambled shRNA empty vector (a gift from David Sabatini; Addgene) was co-transfected into 293T cells with 15 µg psPAX2 packaging plasmid and 6 µg pMD2.G envelope plasmid using calcium phosphate. The culture medium containing lentiviral particles was harvested and centrifuged at 3000× *g* for 5 min to remove cell debris, filtered, and concentrated by ultracentrifugation at 26,000× *g* on a sucrose cushion. hNPCs were transduced with lentiviral particles encoding shcIAP2 or scrambled shRNA, and then puromycin (1 μg/mL) was added to kill non-transfected cells. These cells were then used for subsequent experiments.

### 2.4. Bioluminescence Imaging (BLI) of Grafted hNPCs In Vivo

For BLI in living animals, hNPCs were genetically modified to endogenously express firefly luciferase (Fluc) gene via lentiviral transduction. These Fluc-expressing hNPCs were injected into the HI-injured site of the mice brains, and imaging was conducted using an IVIS Spectrum system (Xenogen Corporation, Alameda, CA, USA). The mice received an intraperitoneal injection of 150 mg/kg D-luciferin (15 mg/mL in phosphate-buffered saline (PBS); Promega, Madison, WI, USA). The BLI signals were acquired as maximum photon flux (photon/s/cm^2^/sr), with the maximum photon flux in the regions of interest being quantified using IGOR software (WaveMetrics, OR, USA).

### 2.5. BV2 and SH-SY5Y Cell Culture

BV2 cells, an immortalized murine microglial cell line, were cultured at 37 °C in DMEM supplemented with 5% fetal bovine serum (FBS; Gibco) and 1% P/S in a humidified incubator with 5% CO_2_ in air. SH-SY5Y cells, a human neuroblastoma cell line, were cultured at 37 °C in DMEM/F12 containing 10% FBS and 1% P/S in a humidified incubator with 5% CO_2_ in air. Cells were seeded into a 10 cm culture dish at a density of 1 × 10^6^ cells per 10 mL culture media. Cells were split when they reached confluence.

### 2.6. Preparation of Conditioned Media

TNF-α was added to the cell culture medium (final concentration: 20 ng/mL) for 24 h. To prepare conditioned media (CM), cells were washed three times with PBS to remove TNF-α from the hNPCs, and they were then seeded at a density of 5 × 10^6^ in culture dishes containing 5 mL of N2 media and incubated for 3 days. The media were then harvested and centrifuged to clarify at 3000× *g* for 5 min. The CM was divided into aliquots and stored at −70 °C until use in assays as TNF-α-pretreated hNPCs-derived CM (TNF-α-hNPC-CM) or non-pretreated hNPC-derived CM (hNPC-CM).

### 2.7. Immunodepletion of CX3CL1 in Conditioned Media

The CX3CL1 was depleted from the culture media using Dynabeads Protein G (Invitrogen, Carlsbad, CA). Briefly, protein G beads were cross-linked with anti-rabbit CX3CL1 immunoglobulin (Santa Cruz Biotechnology, CA, USA). Beads cross-linked with purified normal rabbit IgG (Thermo Scientific, Suwanee, GA, USA) were used as a negative control. The hNPC-CM or TNF-α-hNPC-CM was incubated with anti-CX3CL1 or control IgG beads at room temperature (RT) for 1 h, and then the beads with captured CX3CL1 were removed using a magnet (hNPC-CM-CX3CL1, TNF-α-hNPC-CM-CX3CL1, hNPC-CM-IgG, or TNF-α-hNPC-CM-IgG). The CX3CL1 depleted and IgG depleted conditioned media were collected, and the efficacy of CX3CL1 depletion was confirmed by immunoblot analysis with anti-CX3CL1 antibody.

### 2.8. Co-Culture of Microglia and Neurons

Human neuroblastoma SH-SY5Y cells were plated into poly-L-lysine-coated 24-well dishes at 2 × 10^4^ cells/well with 10 μM retinoic acid, and they were maintained at 37°C in a humidified incubator with 5% CO_2_ in air for 5 d. The media containing retinoic acid was removed, and the SH-SY5Y cells were then co-cultured with microglia in medium alone, hNPC-CM, TNF-α-hNPC-CM, hNPC-CM-CX3CL1, hNPC-CM-IgG, TNF-α-hNPC-CM-CX3CL1, or TNF-α-hNPC-CM-IgG. Then, 100 ng/mL lipopolysaccharides (LPS; Sigma) was added to each medium and incubated at 37 °C for 24 h in a humidified incubator with 5% CO_2_ and air.

### 2.9. Behavioral Assessment

Neurological severity score (NSS), cylinder, and rotarod tests were performed at 1–5, 7, and 9 weeks post-transplantation in the neonatal mice with HI brain injury. All behavioral tests were assessed by an investigator blinded to the experimental groups. Neurological functioning was assessed using the following five reflexes, with each test scored as ‘0′ if the response was normal and ‘1′ if the animal exhibited abnormal reflexes: (1) the ability to straighten its body when lifted to the ground by its tail; (2) the ability to extend its forelimbs when lifted to the ground by its tail; (3) the ability to turn over to a normal position with all four feet on the floor when placed on its side; (4) the ability to raise a paw on a table surface when touching the table edge; (5) and the ability to spread its toes when the board on which it was placed was suddenly bounced.

The cylinder test was used to assess forelimb use and asymmetries during exploratory activity. The mice were placed in an acrylic cylinder (30 cm high, 10 cm diameter) and recorded for 5 min. The numbers of ipsilateral and contralateral paw placements were recorded and analyzed off-line, and the ratio of limb use asymmetry was calculated as follows: (non-impaired forelimb—impaired forelimb)/(non-impaired forelimb + impaired forelimb) × 100.

The rotarod test was used to evaluate motor coordination by testing balance on a rotating rod. Mice were placed on the rotating rod (LSi Letica; Panlab, Barcelona, Spain), and the time-duration over which they remained on the rotating rod was recorded. The revolving speed was gradually accelerated up to 40× *g* and then maintained at this final speed for 5 min. The trial was ended if the animal fell off the rungs or gripped the device and spun around for two consecutive revolutions. The latency of the fall from the rod was recorded as the average of three consecutive trials.

### 2.10. Immunohistochemistry

Mice were deeply anesthetized and transcardially perfused with cold PBS followed by buffered 4% paraformaldehyde. Their brains were removed, post-fixed, cryoprotected in 30% sucrose, and frozen in optimal cutting temperature (OCT) compound (Sakura Finetek, Torrance, CA, USA). The brains were then coronally sectioned at 16 μm with a freezing cryostat (Leica, Wetzlar, Germany). For immunohistochemistry, sections were blocked in 10% normal donkey serum (NDS; Jackson ImmunoResearch, West Grove, PA, USA) and 3% bovine serum albumin (BSA; sigma) in PBS with 0.3% Triton X-100 for 1 h. Sections were then incubated overnight at 4 °C with the primary antibodies for microglia using rabbit anti-ionized calcium-binding adapter molecule 1 (Iba-1, 1:500; Wako, Osaka, Japan), goat anti-Iba-1 (1:100; Abcam, Cambridge, MA), rat anti-CD86 (1:100; BD Biosciences Pharmingen, San Diego, CA, USA), and rabbit anti-arginase 1 antibodies (Arg1, 1:100; Cell Signaling Technology, Inc., Danvers, MA, USA. After being washed three times with PBS, sections were incubated with Alexa-488-conjugated and Alexa-594-conjugated secondary antibodies (1:400; Life Technologies, CA, USA) containing 3% NDS for 70 min at 37 °C in darkness, followed by three PBS washes and mounting using Vectashield mounting medium containing 4′,6-diamidino-2-phenylindole (DAPI; Vector, Burlingame, CA, USA). Immunofluorescence images were captured using a BX51 microscope (Olympus, Center Valley, PA).

### 2.11. Measurement of Infarct Volume

Mice from each group were sacrificed 9 weeks after cell transplantation. The mice were perfused and post-fixed with 4% paraformaldehyde. Their brains were cryoprotected in 30% sucrose, frozen with O.C.T. compound, and cut into a series of 16 μm thick coronal sections using a cryostat. Eight serial coronal sections per mouse (spaced 160 μm apart) were stained with hematoxylin (Vector) and eosin-Y (Sigma), photographed with the Olympus BX51 microscope, and then analyzed with Image J (Broken symmetry software, NIH) by two different persons blind to the experimental groupings. Infarction volumes were calculated as the ([area of the left contralateral hemisphere—area of remaining the right ipsilateral hemisphere]/area of the left contralateral hemisphere) × 100%.

### 2.12. Quantitative Real-Time Polymerase Chain Reaction (qRT-PCR)

Total RNA was extracted from hNPCs, BV2 cells, or tissue dissected from the ipsilateral hemisphere using TRIzol reagent (Molecular Research Center, Cincinnati, OH, USA). RNA was quantified with a spectrophotometer, and 1 μg of isolated RNA was reverse-transcribed into cDNA using a first-strand cDNA synthesis kit (Promega) following the manufacturer’s protocol. qRT-PCR was performed using LightCycler 480 SYBR Green I Master mix (Roche, Mannheim, Germany) on a LightCycler 480 System (Roche) and primers, as previously described [24]. The following primer pairs were used (in pairs, sense, antisense): (1) TNF-α: 5′- CCAGTGTGGGAAGCTGTCTT -3′ and 5′-AAGCAAAAGAGGAGGCAACA-3′; (2) IL1β: 5′-TTCAGGCAGGCAGTATCACTC-3′ and 5′-GAAGGTCCACGGGAAAGACAC-3′; (3) IL6: 5′-TAGTCCTTCCTACCCCAATTTCC-3′ and 5′-TTGGTCCTTAGCCACTCCTTC-3′; (4) iNOS: GGAGTGACGGCAAACATGACT and 5′-TAGCCAGCGTACCGGATGA-3′. (5) CD86: 5′-GCACGTCTAAGCAAGGTCACC-3′ and 5′-TGACATTATCTTGTGATATC-3′. (6) CD206: 5′-CTGTCTCTGTTCAGCTATTG-3′ and 5′-ATGGCACTCCCAAACATAAT-3′; (7) Arginase1: 5′-GCCTCGAGGAGGGGTAGAGA-3′ and 5′-AAAGGAGCTGTCATTAGGGA-3′; (8) CX3CL1: 5′-CGGACAGGATTGACAGATTG-3′ and 5′-CAAATCGCTCCACCAACTAA-3′; (9) CX3CR1: 5′-GTTATTTGGGCGACATTGTGGC-3′ and 5′-GGGCGTAGAAGACGGACAG-3′. (10) GAPDH: 5′-AGGACCAGGTTGTCTCCTGC-3′ and 5′-ACCCTGTTGCTGTAGCCGT-3′.

### 2.13. Western Blotting

hNPCs were lysed in tissue protein extraction reagent (T-PER; Pierce Biotechnology, Rockford, IL, USA) containing protease and phosphatase inhibitors (Sigma), and lysates were centrifuged at 16,000× *g* for 30 min at 4 °C. The Pierce BCA protein assay kit (Pierce Biotechnology) was used for total protein concentrations. The proteins were mixed with 2% β-mercaptoethanol in tricine sample buffer (Bio-Rad, Hercules, CA, USA) and denatured at 95 °C for 10 min. Then, the samples were electrophoresed through 4−15% Mini-PROTEAN TGX precast gels (Bio-Rad) and electrotransferred to nitrocellulose membranes. The membranes were blocked with 5% BSA prepared in Tris-buffered saline containing 0.1% TWEEN 20 (TBST) for 1 h at RT and then incubated overnight at 4°C with the following antibodies: rabbit anti-cleaved caspase-3 (1:1000; Cell Signaling Technology), rabbit anti-CX3CL1 (1:1000) and mouse anti-β-actin (1:2500; Sigma) antibodies. After three washes in TBST, the membranes were hybridized with appropriate horseradish peroxidase-conjugated secondary antibody (1:20000; Jackson ImmunoResearch) for 1 h at RT and washed three times with TBST. Next, the membranes were treated with SuperSignal West Pico Chemiluminescent substrate (Thermo Scientific). The images were observed with an LAS-4000 mini imager (GE Healthcare, Buckinghamshire, UK) and analyzed with MultiGauge software (Fujifilm, Tokyo, Japan).

### 2.14. Terminal Deoxynucleotidyl Transferase-Mediated dUTP Nick End Labeling (TUNEL) Assay

Cells and brain sections treated in various ways were subjected to immunocytochemical detection of apoptosis using an in situ cell death detection kit, FITC or TMR red (Roche), according to the manufacturer’s instructions. After fixation with 4% paraformaldehyde for 30 min and permeabilization with 0.3% Triton X-100 for 15 min, they were incubated with TUNEL reaction mixture at 37 °C for 60 min. The FITC or TMR red-labeled TUNEL-positive cells were imaged using fluorescent microscopy. Stained cells detected green or red fluorescence of apoptotic cells and blue fluorescence of cell nuclei. The proportion of TUNEL-positive cells was determined by analyzing three randomly selected fields per group.

### 2.15. Enzyme-Linked Immunosorbent Assay (ELISA)

One week after transplantation, brains were homogenized with Precellys Lysing kits (Bertin Corporation, Rockville, MD, USA) and Precellys 24 tissue homogenizer (Bertin Corporation) at a ratio of 500 μL to 200 mg tissue in a solution containing PBS and cOmplete ULTRA Tablets protease inhibitor cocktail (Roche). The homogenate was centrifuged at 19,000× *g* for 20 min at RT, and the supernatant was collected. The levels of mouse TNF-α and IL-1β were assayed using mouse TNF-α and IL-1β DuoSet ELISA Development Systems (R&D systems) according to the manufacturer’s instructions.

### 2.16. Cell Viability Assay

Human neuroblastoma SH-SY5Y cells were plated into poly-L-lysine-coated 24-well dishes at 2 × 10^4^ cells/well with 20 mM retinoic acid, and they were maintained at 37 °C in a humidified incubator with 5% CO_2_ in air for 5 d. The media containing retinoic acid were removed, and the SH-SY5Y cells were treated with medium alone, hNPC-CM, or TNF-α-hNPC-CM. After 1 h, cells were challenged with 20-mM glutamate and incubated for another 24 h. Cell viability was measured with a Cell Counting Kit-8 (CCK-8; Dojindo, Kumamoto, Japan), which detects cellular dehydrogenase activity in living cells. CCK-8 reagent was added to each well, and the plate was incubated for an additional 4 h at 37 °C. Then, absorbance of each sample at 450 nm was immediately evaluated using an auto microplate reader (BioTek Instruments; Winooski, VT). All experiments were performed in triplicate and repeated three times independently.

### 2.17. Next-Generation Sequencing (NGS)

hNPCs were stimulated with or without 20 ng/mL TNF-α. After 24 h, total RNA was extracted from hNPCs using TRI reagent (Molecular Research Center). Preparation for RNA library using TruSeq RNA kit (Illumina, San Diego, CA, USA) and Sequencing using HiSeq2000 (Illumina) were carried out by Macrogen Co. (Seoul, Korea) following the manufacturer’s instructions.

All primary analyses of sequence data were performed with the Bowtie2, TopHat2, and Cufflinks packages [26,27,28]. In this study, sequence reads were mapped to the UCSC hg19 reference genome using Bowtie2 and TopHat as previously described [24]. Once the sequence reads were successfully mapped to the reference genome, Cufflinks3 was used for transcript assembly and gene quantification, and then differentially expressed genes (DEGs) were identified using rigorous statistical analysis using Cuffdiff. DEGs were identified by comparing the expression profiles before and after TNF-α treatment.

### 2.18. Statistical Analyses

All statistical analyses were performed using SPSS version 25 (IBM Corp., Armonk, NY, USA). Two-group comparisons were made using the Mann–Whitney U-test, while multiple groups were compared with repeated measures analysis of variance (ANOVA) followed by Bonferroni post hoc analysis for pairwise comparisons between groups. All data are represented as mean ± standard error of the mean (SEM), and *p* < 0.05 was considered to indicate a statistically significant difference.

## 3. Results

### 3.1. TNF-α Pretreatment of hNPCs Increases Survival of Grafted Cells in HI Brain Injury

**Figure 1:**
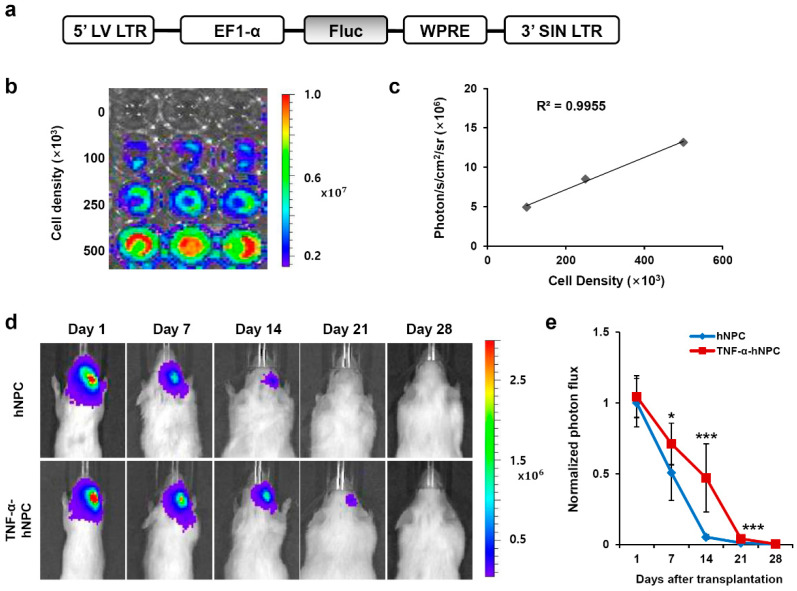
TNF-α pretreatment improved human neural progenitor cell (hNPC) survival in Hypoxic-Ischemic (HI)-injured mouse brain. BLI analysis of transplanted TNF-α-pretreated hNPCs and untreated hNPCs into HI-injured brain. BLI was used to track hNPC survival in vivo for 4 weeks after transplantation. (**a**) Structure of lentiviral vector carrying Fluc under the control of EF-1α promoter. (**b**) In vitro imaging analysis of genetically engineered hNPCs shows increasing Fluc activity with cell density. (**c**) Strong correlation (R2 = 0.9955) was observed between luciferase activity and cell numbers. The data are representative of three independent experiments performed in triplicate. (**d**) Representative images of HI-injured mice transplanted with hNPCs or TNF-α-hNPCs are shown. The color scale bar is in photon/s/cm^2^/sr. (**e**) Bioluminescent signals normalized to signals at day 1 after hNPC injection (n = 13 per group). * *p* < 0.05 and *** *p* < 0.001. Data are presented as the mean ± SEM. Abbreviations: p/s/cm2/sr, photons per second per square centimeter per steridian; LV LTR, lentivirus long-term repeat; EF1-α, EF1-alpha promoter; WPRE, the post-transcriptional regulatory element of woodchuck hepatitis virus; SIN LTR, self-inactivating lentiviral long terminal repeat.

To monitor the survival of transplanted hNPCs in HI-injured brain in vivo, we transduced hNPCs by infecting them with lentiviral particles encoding the Fluc gene (Figure 1a). In vitro image analysis (Figure 1b) demonstrated a linear correlation between cell density and Fluc activity (Figure 1c). The Fluc–expressing hNPCs were injected 3 days after the induction of HI brain injury. For each transplantation group, BLI signals were measured on days 1, 7, 14, 21, and 28 after injection (Figure 1d), and on days 14 and 21 after injection, the TNF-α-hNPC group yielded significantly higher photon flux than the untreated hNPC group. The relative intensity was calculated by normalizing the photon flux at each imaging time point to the photon flux of the hNPC group on the first (day 1) imaging time point. Over 7 days following injection, the hNPC group demonstrated a 50% drop in signal. In comparison, the TNF-α-hNPC group exhibited a 35% drop in signal over the same time period. On the 14th day after injection, BLI signals had markedly decreased in the hNPC group, showing only 5% of the signal shown on the first day, whereas the TNF-α-hNPC group showed 45% of the signal of the first day (Figure 1e). Our results reveal that TNF-α pretreatment improved the viability of hNPCs in HI brain injury.

### 3.2. TNF-α Pretreatment Upregulates cIAP2 Expression and Confers Cytoprotection on hNPCs in HI Brain Injury

**Figure 2:**
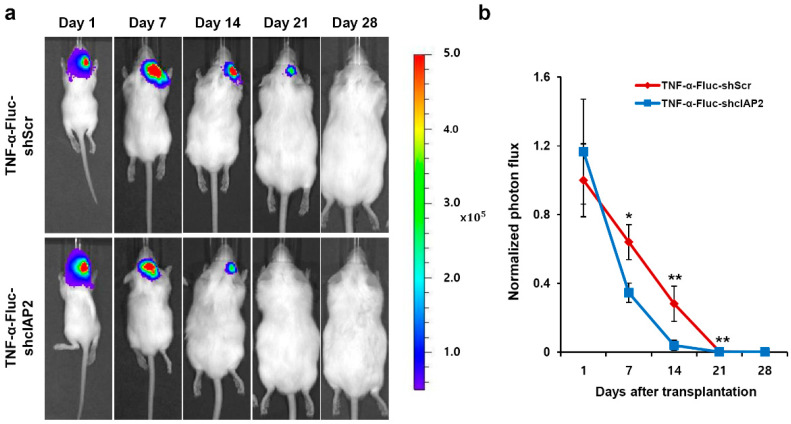
TNF-α pretreatment improved hNPC survival through cIAP2 upregulation. Inhibition of cIAP2 abolishes TNF-α-induced survival of hNPCs in HI brain injury. (**a**) Representative images of HI-injured mice transplanted with TNF-α-Fluc-shScr or TNF-α-Fluc-shcIAP2 are shown. The color scale bar is in photon/s/cm^2^/sr. (**b**) Bioluminescent signals normalized to signals at 1 day after TNF-α-Fluc-shScr cells injection (n = 7 per group). * *p* < 0.05 and ** *p* < 0.01. Data are presented as the mean ± SEM.

We recently showed that TNF-α pretreatment protected hNPCs against the oxygen-glucose deprivation used to mimic ischemic conditions; the pretreatment showed an important preventive effect against oxygen-glucose deprivation-induced apoptosis through cIAP2 upregulation [24]. To investigate whether cIAP2 expression plays an important role in TNF-α-induced protection of hNPCs against HI brain injury, Fluc-expressing hNPCs were infected with lentiviral particles encoding shcIAP2 or scrambled shRNA genes. hNPCs expressing both Fluc and shcIAP2 (TNF-α-Fluc-shcIAP2) or both Fluc and scrambled shRNA (TNF-α-Fluc-shScr) were transplanted into the infarct cavity of each mouse brain 3 days after the induction of HI brain injury. BLI signals were measured at 1, 7, 14, 21, and 28 days after injection in each transplantation group (Figure 2). BLI signals measured 7, 14, and 21 days after injection showed significantly higher photon flux in the TNF-α-Fluc-shScr group than in the TNF-α-Fluc-shcIAP2 group (Figure 2a). Relative intensity was calculated by normalizing the photon flux at each imaging time point to the photon flux on the first (1 d) imaging time point of the TNF-α-Fluc-shScr group. During the first week following injection, the TNF-α-Fluc-shcIAP2 group demonstrated a 70% drop in signal while the TNF-α-Fluc-shScr group exhibited a 36% drop in signal (Figure 2b). On the 14th day after injection, BLI signals had markedly decreased in the TNF-α-Fluc-shcIAP2 group, showing only 3% of the signal shown on the first day, whereas the TNF-α-Fluc-shScr group showed 28% of the signal of the first day. These results suggest that TNF-α-mediated cIAP2 expression is essential to the protection of hNPCs against HI injury.

### 3.3. Transplantation of TNF-α-hNPCs Improves Behavioral Performance and Reduces Infarct Volume After HI Brain Injury

**Figure 3:**
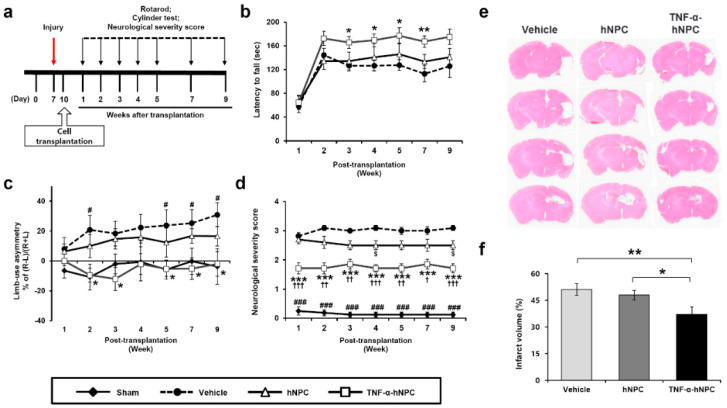
TNF-α-pretreated hNPCs transplantation attenuated the cerebral infarct volume and facilitated functional recovery after HI brain injury. (**a**) Experimental design for behavioral tests. Behavioral tests were performed at 1–5, 7, and 9 weeks post-transplant. Functional recovery after HI brain injury was evaluated using (**b**) a rotarod test, (**c**) a cylinder test, and (**d**) the neurological severity score. (**b**–**d**) * *p* < 0.05, ** *p* < 0.01, and *** *p* < 0.001, for comparisons between TNF-α-hNPC- and vehicle-treated groups; # *p* < 0.05 and ### *p* < 0.001, for comparisons between sham control and vehicle-treated groups; $ *p* < 0.05, for comparisons between hNPC- and vehicle-treated groups; † *p* < 0.05, †† *p* < 0.01, and ††† *p* < 0.001, for comparisons between TNF-α-hNPC- and hNPC-treated groups. (e) Representative image of coronal brain sections (H&E staining) from mice treated with vehicle, hNPCs, and TNF-α-hNPCs at 9 weeks after transplantation. (f) Infarct volume was significantly attenuated in the TNF-α-hNPC group compared with the vehicle and hNPC groups (n = 7 per group). * *p* < 0.05, ** *p* < 0.01. Data are presented as the mean ± SEM.

The effects of TNF-α-hNPC and untreated hNPC transplantation on neurobehavioral deficits induced by HI brain injury were evaluated. Behavioral performance was assessed using rotarod tests, cylinder tests, and neurological severity scores (NSS) at different time points following cells transplantation (Figure 3a). In the rotarod test for assessment of sensorimotor functions, there was no statistical difference between the TNF-α-hNPCs and hNPC groups, but the transplantation of TNF-α-hNPCs significantly increased the duration of the time on the rod at 3–5 and 7 weeks post-transplantation in comparison with the vehicle group (165.74 ± 9.86 vs. 126.70 ± 9.10 sec, *p* < 0.05; 169.45 ± 10.75 vs. 126.64 ± 9.06 sec, *p* < 0.05; 177.02 ± 13.27 vs. 127.52 ± 8.95 sec, *p* < 0.05; and 167.21 ± 9.70 vs. 112.94 ± 13.20 sec, *p* < 0.01, respectively; n = 10–14 per group) (Figure 3b). On the other hand, at all time points, the transplantation of non-treated hNPCs resulted in no significant change in the time on the rod in comparison with the vehicle group (Figure 3b). In the cylinder test for assessment of limb use asymmetry, the vehicle group showed a significant increase in forelimb asymmetry in comparison with the sham control group at 2, 5, 7, and 9 weeks post-transplantation (20.81 ± 9.66 vs. −10.77 ± 8.67, *p* < 0.05; 23.75 ± 10.39 vs. −5.77 ± 6.25, *p* < 0.05; 25.28 ± 9.14 vs. −0.23 ± 8.72, *p* < 0.05; 30.86 ± 8.00 vs. −3.73 ± 11.77, *p* < 0.05, respectively; n = 10−14 per group) (Figure 3c). By contrast, the performance of the TNF-α-hNPC and hNPC groups did not significantly differ from the sham control group, and the TNF-α-hNPC group showed the significant difference compared to the vehicle group at 2, 3, 5, 7, and 9 weeks post-transplantation (Figure 3c). In the NSS for assessment of neurological function, the vehicle group showed significantly higher impairment of neurological function than the sham control group at 1−5, 7, and 9 weeks post-transplantation (2.82 ± 0.12 vs. 0.25 ± 0.14, *p* < 0.001; 3.09 ± 0.09 vs. 0.19 ± 0.10, *p* < 0.001; 3.00 ± 0.01 vs. 0.13 ± 0.09, *p* < 0.001; 3.09 ± 0.09 vs. 0.13 ± 0.09, *p* < 0.001; 3.00 ± 0.13 vs. 0.13 ± 0.09, *p* < 0.001; 3.00 ± 0.13 vs. 0.13 ± 0.09, *p* < 0.001; and 3.09 ± 0.09 vs. 0.13 ± 0.09, *p* < 0.001, respectively; n = 10−14 per group) (Figure 3d). The TNF-α-hNPC group showed significant functional recovery from week 1 through week 9 post-transplantation in comparison with the vehicle group (1.71 ± 0.19 vs. 2.82 ± 0.12, *p* < 0.001; 1.71 ± 0.19 vs. 3.09 ± 0.09, *p* < 0.001; 1.86 ± 0.17 vs. 3.00 ± 0.01, *p* < 0.001; 1.71 ± 0.12 vs. 3.09 ± 0.09, *p* < 0.001; 1.71 ± 0.16 vs. 3.00 ± 0.13, *p* < 0.001; 1.86 ± 0.17 vs. 3.00 ± 0.13, *p* < 0.001; and 1.71 ± 0.16 vs. 3.09 ± 0.09, *p* < 0.001, respectively; n = 10−14 per group) (Figure 3d), and also in comparison with the hNPC group (1.71 ± 0.19 vs. 2.70 ± 0.14, *p* < 0.001; 1.71 ± 0.19 vs. 2.60 ± 0.21, *p* < 0.01; 1.86 ± 0.17 vs. 2.50 ± 0.16, *p* < 0.01; 1.71 ± 0.12 vs. 2.50 ± 0.16, *p* < 0.001; 1.71 ± 0.16 vs. 2.50 ± 0.16, *p* < 0.01; 1.86 ± 0.17 vs. 2.50 ± 0.17, *p* < 0.05; and 1.71 ± 0.16 vs. 2.50 ± 0.16, *p* < 0.001, respectively; n = 10−14 per group) (Figure 3d). However, compared with the vehicle group, the hNPC group showed significant improvement at only 4 and 9 weeks post-transplantation (2.50 ± 0.16 vs. 3.09 ± 0.09, *p* < 0.05 and 2.50 ± 0.16 vs. 3.09 ± 0.09, *p* < 0.05, respectively) (Figure 3d).

Infarct volume was measured in coronal sections by hematoxylin and eosin (H&E) staining at 9 weeks after transplantation by subtracting the viable tissue volume in the ipsilateral hemisphere from that of the contralateral hemisphere. The percentages of infarct volume in the TNF-α-hNPC, hNPC, and vehicle groups were 37.1% ± 4.20%, 47.9% ± 2.66%, and 51.0% ± 3.28%, respectively, as determined by H&E staining (Figure 3e,f). The infarct size in the TNF-α-hNPC group was about 14% lower than that in the vehicle group, but there was no significant difference in infarct volume between the hNPC and vehicle groups (Figure 3f).

### 3.4. Transplantation of TNF-α-hNPCs Decreases The Number of TUNEL-Positive Cells and The Expression Levels of Cleaved Caspase 3 in HI Brain Injury

**Figure 4:**
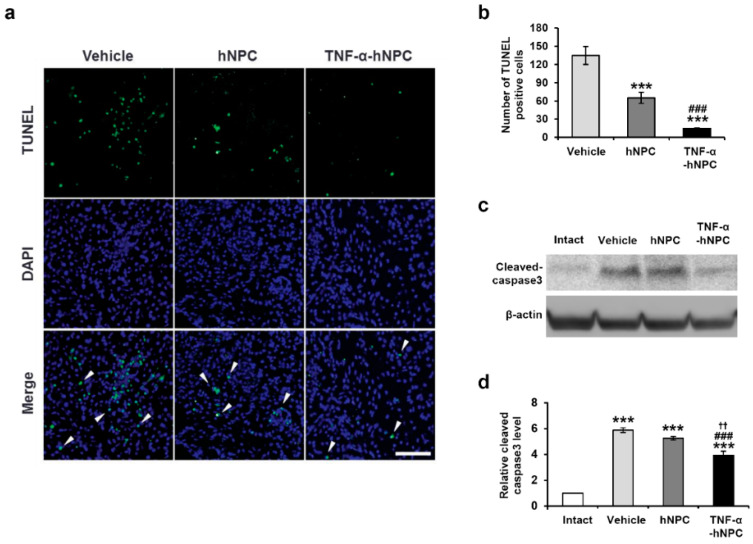
TNF-α-pretreated hNPCs induced cerebral neuroprotection after HI brain injury. (**a**) Representative images of TUNEL+ cells within the ipsilateral cortex in the vehicle-, hNPC-, and TNF-α-hNPC-injected mice 4 weeks after HI brain injury. Scale bars, 50 μm. (**b**) Quantitative analysis of TUNEL+ apoptotic cells (arrowheads in a; n = 3 per group). *** *p* < 0.001 vs. Vehicle and ### *p* < 0.001 vs. hNPC. (**c**) Protein levels of cleaved caspase-3 were determined from mice treated with vehicle, hNPCs and TNF-α-hNPCs. β-actin was used as an internal control. (**d**) Quantification of cleaved caspase-3 expression (n = 3 per group). *** *p* < 0.001 vs. Sham, ### *p* < 0.001 vs. Vehicle and †† *p* < 0.01 vs. hNPC. Data are presented as the mean ± SEM.

To investigate whether transplantation of TNF-α-hNPCs could facilitate amelioration of HI brain injury, a TUNEL analysis was performed at 4 weeks after transplantation (Figure 4a). TUNEL+ staining of dying brain cells with fragmented DNA was significantly lower in the TNF-α-hNPC group and hNPC group in comparison with the vehicle group (14.43 ± 1.29 vs. 134.67 ± 14.72, *p* < 0.001 and 65.00 ± 9.22 vs. 134.67 ± 14.72, *p* < 0.001, respectively) (Figure 4a,b). The TNF-α-hNPC group also showed a significant reduction in apoptotic cells in comparison with the hNPC group (14.43 ± 1.29 vs. 65.00 ± 9.22, *p* < 0.001) (Figure 4a,b).

Next, to investigate the impact of donor stem cells on neuronal death 7 days after injection, we quantified the expression levels of cleaved caspase-3. Caspase-3 is a cysteine protease that is cleaved in neurons within the HI core and penumbra. We used Western blotting to quantify the expression levels of cleaved caspase-3 in the ipsilateral hemispheres of HI-injured brains. Caspase-3 activity in the ipsilateral side of the vehicle, hNPC, and TNF-α-hNPC groups was significantly higher than in the sham group (Figure 4c,d). This elevated caspase-3 activity was partially inhibited in the TNF-α-hNPC group compared with the vehicle, but not in the hNPC group (Figure 4c,d). These results indicate that transplantation of TNF-α-pretreated hNPCs exerted a neuroprotective effect in vivo.

### 3.5. Transplantation of TNF-α-hNPCs alters the M1/M2 Phenotype of Microglia and Decreases Neurotoxicity in HI Brain Injury

**Figure 5:**
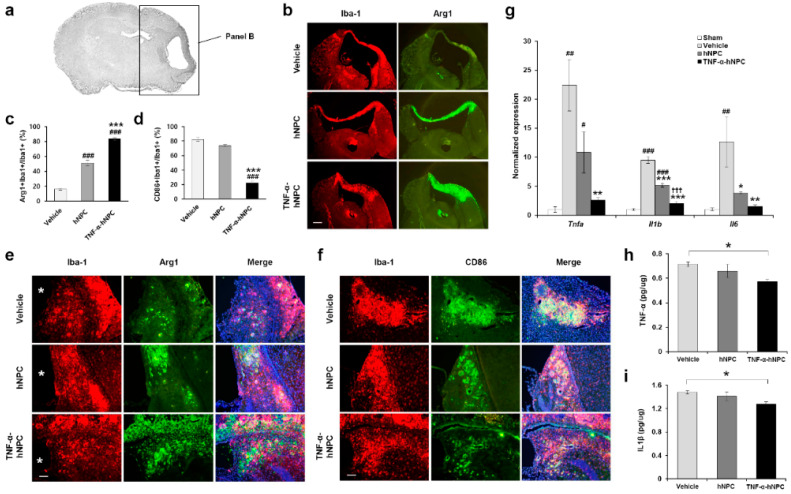
TNF-α-pretreated hNPCs altered the ratio of M1/M2 phenotype of microglia and decreased proinflammatory cytokines in HI brain injury. (**a**) A low-magnification view of HI-injured brain. The black box represents the ipsilateral hemisphere of HI-injured brain at 2 weeks post-transplant. (**b**) The ipsilateral hemisphere co-stained for Arg1 (M2 marker, green) and Iba-1 (red). Scale bar, 50 μm. (**c**) Quantification of the percentage of Arg1+/Iba-1+ cells (n = 3 per group). (**d**) Quantification of the percentage of CD86+/Iba-1+ cells (n = 3 per group). (**e**) A higher magnification image of Arg1/Iba1-positive cells. An asterisk indicates the infarct area. Scale bar, 50 μm. (**f**) Representative images of Iba1 (red) and CD86 (green) immunostaining 2 weeks after HI brain injury. Scale bar, 50 μm. ### *p* < 0.001 vs. vehicle, *** *p* < 0.001 vs. hNPCs. (**g**–**i**) Effects of TNF-α-pretreated hNPCs on modulation of inflammatory responses at 1 week post-transplant. (**g**) qRT-PCR for relative expression levels of mouse cytokine mRNA in the ipsilateral hemisphere of HI brain (n = 3–5 per group). # *p* < 0.05, ## *p* < 0.01, ### *p* < 0.001 vs. Sham; * *p* < 0.05, ** *p* < 0.01, *** *p* < 0.001 vs. vehicle; ††† *p* < 0.001 vs. hNPC. (**h**,**i**) ELISA for mouse TNF-α (**h**) and IL-1β (**i**) proteins in the ipsilateral hemisphere of HI brain injury (n = 7–8 per group). * *p* < 0.05. Data are presented as the mean ± SEM.

Protection against HI brain injury was more evident in the TNF-α-hNPC group with improved behavioral performance (Figure 3b–d) and reduced infarct size (Figure 3e,f) being shown. As the major pathological mechanisms of early brain injury after HI insult are said to be hypoxia-ischemia and inflammation [29,30], and microglia activation is thought to contribute to inflammation in HI brain injury, we investigated the effects of TNF-α-hNPCs on the functional status of microglia. To evaluate the possible effects of hNPC transplantation on M1/M2 phenotypic transition of microglia, double staining of CD86 (a marker of M1 phenotype microglia) or Arg1 (a marker of M2 phenotype microglia) with Iba1 was performed at 2 weeks after transplantation. The fluorescent immunostaining clearly showed increased M2 but reduced M1 microglia phenotypes in the hNPC and TNF-α-hNPC groups compared with the vehicle group, as evidenced by the increased Arg1+/Iba1+ (51.1% ± 3.6% vs. 15.9% ± 1.4% and 83.6% ± 1.8% vs. 15.9% ± 1.4%, *p* < 0.001, respectively; n = 3 per group) (Figure 5b,c,e) and reduced CD86+/Iba1+ microglia cells (73.8% ± 1.3% vs. 82.3% ± 2.8% and 22.2% ± 2.1% vs. 82.3% ± 2.8%, *p* < 0.001, respectively; n = 3 per group) (Figure 5d,f). The CD86+/Iba1+ microglia percentage of the TNF-α-hNPC group was significantly lower than that of the hNPC group (22.2% ± 2.1% vs. 73.8% ± 1.3%, *p* < 0.001; n = 3 per group) (Figure 5d,f), and the Arg1+/Iba1+ percentage of the TNF-α-hNPC group was significantly higher than that of the hNPC group (83.6% ± 1.8% vs. 51.1% ± 3.6%, *p* < 0.001; n = 3 per group) (Figure 5b,c,e). Next, the effects of TNF-α pretreated-hNPCs on expression of the proinflammatory mediators were tested to confirm the M1/M2 phenotypic changes mentioned above. Proinflammatory mediator expression is increased immediately after injury. Microglia-mediated neurotoxicity is induced through the release of pro-inflammatory cytokines such as TNF-α, IL-1β, and IL-6 [31,32]. Therefore, the expression of cytokines was examined at 1 week after transplantation using qRT-PCR for mRNA level and ELISA for protein level (Figure 5g–i). Compared with the sham control group, the mRNA levels of *Tnfa*, *Il1b,* and *Il6* were significantly higher in the vehicle group, and the mRNA levels of *Tnfa* and *Il1b* were significantly higher in the hNPC group. However, there was no significant change between the TNF-α-hNPC and sham control groups. Compared with the vehicle group, the mRNA levels of *Tnfa*, *Il1b,* and *Il6* were significantly lower in the TNF-α-hNPC group (Figure 5g), and the mRNA levels of *Il1b* and *Il6* were significantly lower in the hNPC group, although they were not as low as in the TNF-α-hNPC group. The mRNA levels of *Il1b* were markedly reduced in the TNF-α-hNPC group in comparison with the hNPC group (Figure 5g). The protein levels of mouse TNF-α and IL-1β were assayed by ELISA at 1 week after transplantation, and the results showed that the expression levels of mouse TNF-α (0.58 ± 0.01 pg/μg vs. 0.71 ± 0.02 pg/μg, *p* < 0.05) (Figure 5h) and mouse IL-1β (1.28 ± 0.04 pg/μg vs. 1.48 ± 0.03 pg/μg, *p* < 0.05) (Figure 5i) were significantly lower in the TNF-α-hNPC group than in the vehicle group. The protein levels of mouse TNF-α and IL-1β were not significantly different between the vehicle and hNPC groups.

### 3.6. TNF-α Pretreatment Reduces Neuronal Cell Death in Neuron-Activated Microglia Co-Culture

**Figure 6:**
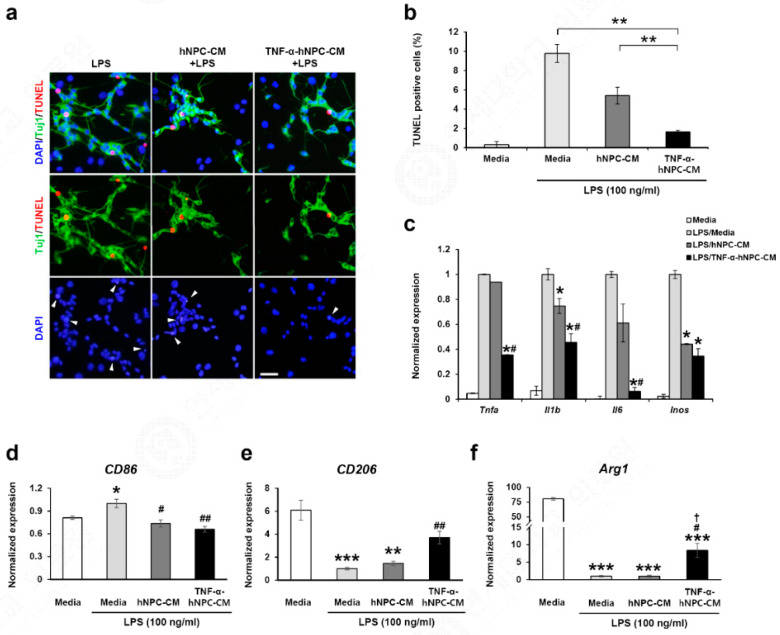
TNF-α-pretreated hNPC-CM prevented neuronal damage induced by reactive microglia in neurons and microglia co-cultures. (**a**) Neuron and microglia co-cultures were treated with media, hNPC-CM, or TNF-α-hNPC-CM in the presence or absence of 100 ng/mL LPS. Cell-specific markers and a separate nuclear stain were combined with a TUNEL assay. TUNEL: TMR red (red), neuron-specific marker: Tuj1 (green), nucleus-specific marker: DAPI (blue). The total cell number per field was established by counting individual nuclei (blue). Turning the laser channel on/off for nuclear stain (blue) allowed easy detection of overlap between cell-specific (green) and TUNEL (red) markers (middle panel). Scale bars, 25 μm. The nuclei indicated by arrowheads overlap with the TUNEL+ apoptotic cells (lower panel). (**b**) Quantitative analysis of TUNEL assay. TUNEL-positive cells were counted from four images of randomly selected fields per group, and at least 100 cells were counted per field. Data are presented as the means ± SEM from three independent experiments performed in triplicate. ** *p* < 0.01. (**c**) Evaluation of pro-inflammatory cytokines in BV2 cells after 24 h. The expression of *Tnfa*, *Il1b*, *Il6*, and *Inos* was assessed by qRT-PCR. (n = 6 per group). * *p* < 0.05 vs. LPS/Media; # *p* < 0.05 vs. LPS/hNPC-CM. (**d**–**f**) Evaluation of M1/M2 polarization markers. The microglial phenotypes of BV2 co-cultured with media, hNPCs, or TNF-a-hNPCs were assessed by qRT-PCR of the M1 marker CD86 (**d**), and the M2 markers CD206 (**e**) and Arg1 (**f**) (n = 3 per group). * *p* < 0.05, ** *p* < 0.01, and *** *p* < 0.001 vs. Media only; # *p* < 0.05 and ## *p* < 0.01 vs. LPS/Media; † *p* < 0.05 vs. LPS/hNPC-CM. Data are presented as the mean ± SEM.

To directly observe the effect of TNF-α pretreatment on the interactions of activated microglia and neurons, a co-culture system including neurons and microglia was used, with the microglia cells being stimulated with LPS. Human neuroblastoma SH-SY5Y cells were initially differentiated by treatment with retinoic acid. The retinoic acid-containing media were then removed, and the SH-SY5Y cells were co-cultured with BV2 cells in medium alone, medium with LPS, hNPC-CM with LPS, and TNF-α-hNPC-CM with LPS for 24 h (Media, Media+LPS, hNPC-CM+LPS, and TNF-α-hNPC-CM+LPS groups, respectively), after which neuronal cell death was assessed by TUNEL staining. Microscopy images of mixed cultures stained with TUNEL and cell-specific markers showed that most of the cells that had died in response to LPS-induced reactive microglia were SH-SY5Y cells (Figure 6a). Significant increases in SH-SY5Y cell apoptosis occurred consistently in cell cultures when LPS was included in the culture conditions. The hNPC-CM+LPS and TNF-α-hNPC-CM+LPS groups showed reduced neuronal apoptosis compared with the Media+LPS group, and the TNF-α-hNPC-CM+LPS group showed significantly lower neuronal cell death compared with the hNPC-CM+LPS group (Figure 6a,b). Next, we investigated the effects of TNF-α pretreatment of hNPCs on the modulation of inflammatory responses in vitro, first assessing the expression of inflammatory cytokines by LPS-stimulated microglia. As shown in Figure 6c, the *Tnfa*, *Il1b*, *Il6*, and *Inos* mRNA levels of BV2 cells activated with 100 ng/mL LPS (LPS/Media) were elevated. Compared with the LPS/Media group, *Il1b* and *Inos* mRNA expression in the activated BV2 cells with hNPC-CM (LPS/hNPC-CM) was significantly decreased. There was no difference in *Tnfa* and *Il6* between the LPS/Media and LPS/hNPC-CM groups. The mRNA levels of *Tnfa*, *Il1b*, *Il6*, and *Inos* of the BV2 cells in the LPS/TNF-α-hNPC-CM group were significantly lower than those in the LPS/Media group. Additionally, the mRNA levels of *Tnfa*, *Il1b,* and *Il6* in the LPS/TNF-α-hNPC-CM group were lower than those in the LPS/hNPC-CM group (Figure 6c). We then examined the expression of markers for M1 (CD86) and M2 (Arg1, CD206) microglia in BV2 cells treated with LPS/Media, LPS/hNPC-CM, or LPS/TNF-α-hNPC-CM. The expression of CD86 mRNA in the BV2 cells was significantly downregulated in the LPS/TNF-α-hNPC-CM group and the LPS/hNPC-CM group. The CD86 mRNA levels of the LPS/TNF-α-hNPC-CM group were lower than those in the LPS/hNPC-CM group (Figure 6d). The expression of Arg1 and CD206 mRNAs, which was reduced by LPS in the BV2 cells, was significantly upregulated in the LPS/TNF-α-hNPC-CM group, whereas significant upregulation was not observed in the LPS/hNPC-CM group (Figure 6e,f). Our results suggest that factors secreted by the TNF-α pretreated-hNPCs protected neurons through regulation of microglia.

### 3.7. TNF-α Pretreatment Upregulated CX3CL1 Expression and Induced Neuroprotection via CX3CL1-Dependent Microglia Polarization

**Figure 7:**
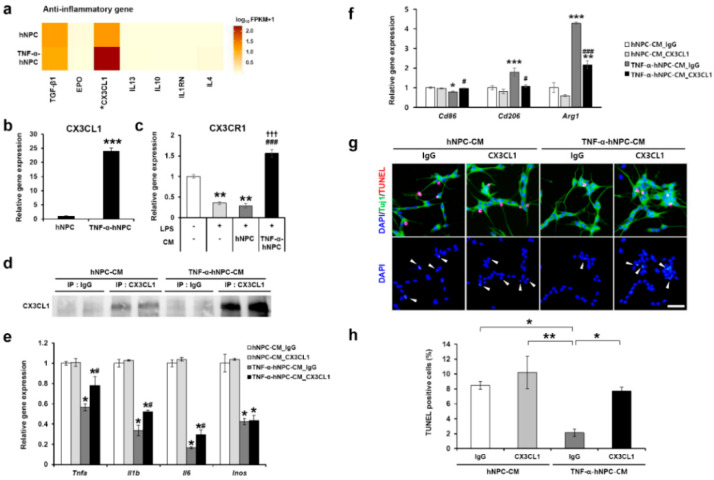
TNF-α-induced CX3CL1 affected microglia polarization and prevented neuronal damage in neuron-activated microglia co-cultures. (**a**) Heatmap of RNA-seq data analysis of transcripts encoding genes associated with inflammatory biological pathways. The heatmap was generated using the cummeRbund R package and converted to log10 FPKM values. An asterisk in heatmaps denotes CX3CL1 gene discussed in the text. (**b**) qRT-PCR analysis of CX3CL1, with significantly increased expression by TNF-α treatment in hNPCs. *** *p* < 0.001. (**c**) qRT-PCR analysis of CX3CR1 in BV2 cells. ** *p* < 0.01 vs. Media only; ### *p* < 0.001 vs. LPS/Media; ††† *p* < 0.001 vs. hNPC-CM. (**d**) CM from hNPCs or TNF-α-hNPC was depleted with 5 µg/mL of monoclonal anti-rabbit CX3CL1 antibody or rabbit IgG. The CM treated with anti-CX3CL1 or normal IgG were immunoblotted with anti-CX3CL1. (**e**) Depletion of CX3CL1 in TNF-α-hNPC-CM significantly inhibited the decrease of pro-inflammatory genes compared with depletion of IgG in TNF-α-hNPC-CM, as quantified by qRT-PCR. f qRT-PCR was performed to quantify the mRNA expression of CD86, CD206, and Arg1 in BV2 cells. (**e**, **f**) * *p* < 0.05, ** *p* < 0.01, and *** *p* < 0.001 vs. hNPC-CM-IgG; # *p* < 0.05, ### *p* < 0.001 vs. TNF-α-hNPC-CM-IgG. (**g**) Cell-specific markers and separate nuclear stain were combined with a TUNEL assay. TUNEL: TMR red (red), neuron-specific marker: Tuj1 (green), nucleus-specific marker: DAPI (blue). Total cell numbers per field were established by counting individual nuclei (blue). The nuclei indicated by arrows (lower panel) overlap with TUNEL+ apoptotic cells (upper panel). Scale bars, 25 μm. (**h**) Quantitative analysis of the TUNEL assay. TUNEL-positive cells were counted in four images of randomly chosen fields per group, and at least 100 cells were counted per field. Data are presented as the means ± SEM from three independent experiments performed in triplicate. * *p* < 0.05 and ** *p* < 0.01.

We carried out RNA-seq analysis to investigate the influence of TNF-α on hNPC gene expression and assess the expression of anti-inflammatory-related genes in stem/progenitor cells. The expression of CX3CL1 in RNA-seq was higher in TNF-α-pretreated hNPCs than in untreated hNPCs (Figure 7a), and in vitro qPCR analysis showed that CX3CL1 expression was increased 25-fold compared with non-pretreated hNPCs (Figure 7b). While the treatment of BV2 cells with LPS reduced the expression of the CX3CL1 receptor CX3CR1 in the cells, the increased expression of CX3CR1 was observed in TNF-α-hNPC-CM+LPS group, but not in the hNPC-CM+LPS group (Figure 7c). In vitro tests were performed to determine whether TNF-α-induced CX3CL1 decreased the expression level of proinflammatory cytokines in activated microglia. The immunodepletion technique was utilized to remove CX3CL1 polypeptides from hNPC-CM or TNF-α-hNPC-CM. As shown in the Western blot data (Figure 7d), CX3CL1 polypeptides were effectively removed by incubation with anti-CX3CL1 cross-linked beads, whereas beads cross-linked with normal IgG were unable to deplete CX3CL1 from the CM. Depletion of CX3CL1 by treating TNF-α-hNPC-CM with anti-CX3CL1 antibody (TNF-α-hNPC-CM-CX3CL1) for 24 h resulted in the upregulation of pro-inflammatory gene expression in LPS-stimulated microglia in comparison with depletion of IgG by treating TNF-α-hNPC-CM with anti-IgG antibody (TNF-α-hNPC-CM-IgG). Treatment of BV2 cells with TNF-α-hNPC-CM significantly decreased the expression levels of pro-inflammatory genes including *Tnfa*, *Il1b*, *Il6*, and *iNOS* (Figure 6c and Figure 7e). Depletion of CX3CL1 in this TNF-α-hNPC-CM reduced the extent of this decrease, at least for *Tnfa*, *Il1b*, and *Il6* (Figure 7e). Moreover, we examined the possible effects of CX3CL1 on M1/M2 phenotypic transition. In BV2 cells, the expression of CD86 mRNA was significantly upregulated, and the expression of Arg1 and CD206 mRNA was significantly downregulated in the TNF-α-hNPC-CM-CX3CL1 compared with TNF-α-hNPC-CM-IgG (Figure 7f).

Next, to evaluate whether TNF-α induced CX3CL1 could reduce neuronal cell death under stress in vitro, we used a co-culture system, including neurons and microglia, and the immunodepletion technique for the removal of CX3CL1 polypeptides from the hNPC-CM or TNF-α-hNPC-CM. The TNF-α-hNPC-CM-IgG group showed reduced neuronal apoptosis compared with the hNPC-CM-IgG, hNPC-CM-CX3CL1, and TNF-α-hNPC-CM-CX3CL1 groups. The TNF-α-hNPC-CM-CX3CL1 group significantly inhibited the decrease of neuronal cell death compared with the TNF-α-hNPC-CM-IgG group (Figure 7g,h). These results suggest that TNF-α-induced CX3CL1 expression could have an anti-inflammatory effect during HI brain injury.

### 3.8. TNF-α Pretreatment Protects Neurons Under Glutamate Stress via Secretion of Neurotrophic Factors

**Figure 8:**
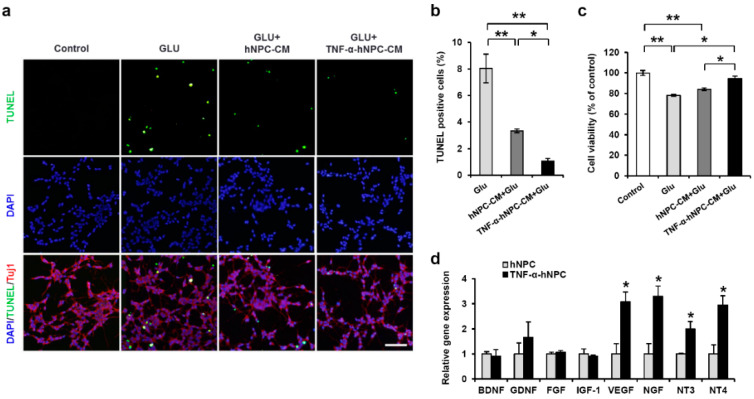
Secreted factors released from TNF-α pretreated hNPCs promoted survival of neuronal cells exposed to glutamate. (**a**) A TUNEL assay was used to measure apoptosis in neurons after glutamate exposure with hNPC-CM and TNF-α-hNPC-CM for 24 h. (**b**) Quantitative analysis of the TUNEL assay. TUNEL-positive cells were counted in four images of randomly chosen fields per group, and at least 100 cells were counted per field. Data are presented as the means ± SEM from three independent experiments performed in triplicate. (**c**) The viability of SHSY5Y cells was assessed by a CCK-8 assay after glutamate exposure with hNPC-CM and TNF-α-hNPC-CM for 24 h. (**d**) qRT-PCR was performed to quantify the mRNA expression of neurotrophic factors in hNPCs and TNF-α-hNPCs. * *p* < 0.05 and ** *p* < 0.01. Data are presented as the mean ± SEM.

As NPCs can regulate the release of many neurotrophic factors and other soluble molecules that modify the secretion of oxidative reaction and inflammatory mediators [33,34,35], we tested whether TNF-α altered their expression in hNPCs in vitro. Significantly higher gene expressions of vascular endothelial growth factor (*VEGF*), nerve growth factor (*NGF*), neurotrophin 3 (*NT3*), and neurotrophin 4 (*NT4*) were detected in the TNF-α-pretreated hNPCs than in the non-pretreated hNPCs (Figure 8d). To assess the protective effect of neurotrophic factors secreted by TNF-α pretreatment against glutamate toxicity, SH-SY5Y cells were incubated in medium alone, medium with glutamate, hNPC-CM with glutamate, or TNF-α-hNPC with glutamate for 24 h (control, Glu, hNPC-CM+Glu, and TNF-α-hNPC-CM+Glu groups, respectively). The hNPC-CM+Glu and TNF-α-hNPC-CM+Glu groups showed reduced neuronal apoptosis compared with the Glu group. In this study, glutamate exposure resulted in significant toxicity in SHSY5Y cells (Figure 8a–c), but treatment of the cells with TNF-α-hNPC-CM+Glu significantly prevented glutamate-induced loss of cell viability in comparison with treatment with hNPC-CM+Glu (Figure 8b). These results suggest that the increased secretion of neurotrophic factors induced by pretreatment of hNPCs with TNF-α could have a neuroprotective effect during HI brain injury.

## 4. Discussion

In this study, we demonstrated that TNF-α pretreatment enabled hNPCs to survive longer after transplantation into HI brain injury and improved the neuroprotective effect of hNPC transplantation after focal HI brain injury. We found that a cIAP2-targeting shRNA abolished TNF-α-induced cytoprotection against HI-injured brain in neonatal mice. In addition, we also showed that the neuroprotective effects of TNF-α pretreated hNPCs are associated with anti-inflammatory effects by modulating microglia phenotypes in a CX3CL1-dependent manner, and with neurotropic factor effects by secreting *VEGF*, *NGF*, *NT3*, and *NT4*. Furthermore, transplantation of TNF-α-hNPCs led to better recovery of behavioral performance and reduction of infarct size. Therefore, we conclude that the prevention of apoptosis and enhancement of neuroprotective effects of stem/progenitor cells without the need for genetic manipulation is a beneficial and safe approach for improving the therapeutic efficacy of hNPC transplantation in HI brain injury.

Several studies have shown that TNF-α affects the survival, differentiation, and proliferation of neural stem/progenitor cells, and contributes to neuroprotection and tissue regeneration in stroke, status epilepticus, and inflammation [21,22,23,36]. The sensitivity of diverse cell types to various TNF-α doses has been assessed under different experimental conditions [37,38]. Our previous report demonstrated that TNF-α pretreatment at a concentration of 20 ng/mL showed the highest survival rate of hNPCs in ischemia-related in vitro conditions [24], and we therefore mainly used this concentration in this study.

In the current study, TNF-α-pretreated-hNPCs and non-pretreated hNPCs were injected 3 days after induction of HI brain injury. In animal models of HI brain injury, stem cells have been transplanted between the first hour and 7 days after brain injury, with different degrees of beneficial outcome [14,39,40,41]. Most of these studies have a variety of types, including transplant cell types, injury models, evaluation methods, etc., making it difficult to determine the best time for stem/progenitor cell therapy. The function of stem/progenitor cell therapy may vary at different stages of brain injury. According to a previous report on NSC transplantation, engraftment of stem cells was maximal 3–7 days after injury [42]. We decided to transplant 3 days after injury, but since we did not compare the therapeutic efficiencies at time of transplantation after injury, it is difficult to directly compare our study with previous studies. Our previous report also showed that TNF-α pretreatment increased the expression of the survival-related gene *cIAP2* in hNPCs [24]. cIAP2 plays an important role in cell death by suppressing apoptosis [43,44]. Studies have shown that ischemic preconditioning protected against cerebral ischemia by inhibiting caspase-3 and preserving cIAP2 in the hippocampus [45], and cIAP2-null macrophages are highly sensitive to apoptosis in an LPS-induced proinflammatory environment [46]. These findings suggest that the TNF-α pretreatment, which induced cIAP2 expression, conferred cytoprotection after HI brain injury. To demonstrate that cIAP2 protects hNPCs under HI brain injury, we used a lentivirus-mediated shRNA system to knockdown cIAP2 in hNPCs, and BLI to measure hNPC survival. Our results indicate that cIAP2 could be a potential therapeutic target for the survival of engrafted stem/progenitor cells, and that, consequently, TNF-α pretreatment protects transplanted hNPCs in HI-injured brain.

Another finding of this study is that TNF-α pretreatment enhances the neuroprotective effect of hNPC transplantation after HI brain injury. Cerebral ischemia induces a strong inflammatory response characterized by rapid microglial activation, which increases oxidative stress [47] and inflammatory mediators [48,49]. In addition, this activation is correlated with increased neuronal death. In this study, we found that mRNA levels of pro-inflammatory cytokines such as *Tnfa*, *Il1b*, and *Il6* were markedly reduced after transplantation of TNF-α-hNPCs, and that the M1/M2 ratio of microglia in the ipsilateral injured cortex was significantly decreased in the TNF-α-hNPCs injection group in comparison with the vehicle injection group, as evidenced by the decreased CD86+/Iba1+ and the increased Arg1+/Iba1+ microglia cells. Microglia can be activated in two states, a classical pro-inflammatory state (M1) and an alternative anti-inflammatory state (M2), with these two phenotypes playing a contrasting role during tissue damage. The pro-inflammatory M1 phenotype secretes high levels of pro-inflammatory cytokines that can cause cell death and tissue damage. On the contrary, the M2 phenotype is associated with neuroprotective effects in neurodegenerative diseases of the brain [50,51]. Imbalance in the M1 and M2 phenotypes, especially the lack of a proper M2 phenotype, is associated with neurodegeneration including spinal cord injury, traumatic brain injury, ischemic stroke, and Alzheimer’s disease [52,53]. Conversely, enhanced M2 polarization is generally neuroprotective in several other neuropathy models [53,54]. Another study showed that, after injury, CD86-positive cells were increased, and the relative proportion of CD206-positive cells was decreased, indicating that HI brain injury might promote M1 polarization and reduce M2 activation [55]. Consistent with this, in the current neuron-microglia co-culture study, LPS-stimulated BV2 cells significantly increased the M1 gene *Cd86* and decreased M2 genes including *Cd206* and *Arg1*. TNF-α-hNPC-CM significantly decreased *Cd86* and increased *Cd206* and Arg1 in comparison with the media and hNPC-CM groups. By using RNA-seq analysis and qRT-PCR, we found that CX3CL1, an important factor for regulation of microglia, was increased in hNPCs by TNF-α treatment. Previous studies have shown that gene expression of CX3CL1 is regulated by TNF-α in other cell types [56], that CX3CL1 inhibits neuronal death by reducing the production of pro-inflammatory cytokine via inhibition of LPS-induced microglial activation [57,58,59], and that stem cells genetically modified to overexpress CX3CL1 exhibited enhanced neuroprotective effects, and reduced ischemia-induced cerebral infarct size and neurological deficits [60,61]. These findings suggest that, in this study, CX3CL1 contributed to the TNF-α-mediated neuroprotective effects. Using conditioned media from hNPCs and a co-culture system of microglia and neuronal cells in vitro, it was shown that CX3CL1 depletion from TNF-α-hNPC-CM suppresses the reduction of proinflammatory mediators and neuronal damage mediated by TNF-α-hNPC-CM. We also found that, in comparison with IgG depletion from TNF-α-hNPC-CM, CX3CL1 depletion from TNF-α-hNPC-CM resulted in specific significant upregulation of CD86 expression and downregulation of CD206 and Arg1 expression in microglia. A previous study demonstrated that CX3CL1 could induce M2 polarization of macrophages with decreased expression of CD86 [62], and we found a significant increase of its receptor CX3CR1 levels reduced by LPS in microglia with TNF-α-hNPC-CM. The main effect of CX3CR1 binding on the unique ligand CX3CL1 released by neurons is to control microglial activation. Disruption of the CX3CL1–CX3CR1 axis leads to dysregulation of microglial and neuronal damage in various animal models of neurodegenerative diseases such as Parkinson’s disease, ischemic stroke, Alzheimer’s disease, and mild traumatic brain injury [63,64,65,66]. The deletion of CX3CR1 expression could promote microglial activation, and enhancement of CX3CR1 expression could protect against microglial neurotoxicity in the model of Parkinson’s disease and a transgenic model of amyotrophic lateral sclerosis [67]. The CX3CL1–CX3CR1 axis may play a crucial role in immunoregulation in various neurodegenerative diseases. Our results indicate that the neuroprotective effects of TNF-α-hNPCs in HI brain injury could be provided by stimulation of microglial CX3CR1 expression, as well as direct release of CX3CL1. This is consistent with recent studies showing that mesenchymal stem cells have beneficial effects by altering the M1-M2 transition towards the neuroprotective phenotype through the release of chemokine CX3CL1 and its interaction with microglial CX3CL1 receptors (CX3CR1) [68]. Thus, TNF-α-pretreated hNPC-derived CX3CL1 play an important role in improving the neuroprotective effect in mice after HI brain injury by regulating the CX3CL1–CX3CR1 axis. However, several studies reported that CX3CL1–CX3CR1 signaling contributes positively to neuroprotective as well as detrimental role in the course of the different neurological diseases as noted above [67,69]. The difference of CX3CL1 concentration/expression, distribution of CX3CR1 on the cell surface, or selective activation of CX3CL1–CX3CR1 downstream signaling pathways in different disease models may be the cause of the difference in biological efficacy. These findings indicate that the biological function of CX3CL1–CX3CR1 has a complex regulatory mechanism and needs to be further investigated.

In the present study, we also evaluated the ability of TNF-α-pretreated hNPCs to antagonize glutamate-mediated excitotoxicity in differentiated SH-SY5Y cells. Glutamate excitotoxicity is associated with many neurological diseases, including HI injury, epilepsy, and other neurodegenerative disorders responsible for neuronal cell death [70]. The expressions of multiple neurotrophic factors, especially VEGF, NGF, NT3, and NT4 that are known to be important for neuroprotection [71,72,73], were increased after TNF-α pretreatment of hNPCs. These neurotrophic factors are important regulators of neuronal development, differentiation, proliferation, and maturation in the peripheral and central nervous system [74,75]. Experimental animal models have shown that these neurotrophic factors can be effective in helping to prevent neuronal loss and brain damage after HI brain injury [74,76,77]. In agreement with these previous studies on the neuroprotective role of neurotrophic factors, our study showed that secretion of neurotrophic factors through altered expression by TNF-α pretreatment reduced glutamate-induced neuronal apoptosis. Although it may be necessary to more closely study the effect of these factors on neuroprotection in the transplantation of TNF-α pretreated hNPCs, many of the changes observed after TNF-α pretreatment are associated with several mechanisms of interaction related to the neuroprotection and functional benefits that occur after transplantation. Taken together, our results show that TNF-α-pretreated hNPCs improve their survival upon implantation into HI-injured brain and exert neuroprotection through multiple mechanisms such as modulation of microglial polarization, inhibition of proinflammatory cytokines, and upregulation of neurotrophic factors.

## 5. Conclusions

In summary, we have shown that pretreatment of hNPCs with TNF-α prior to transplantation into HI-injured brain enhanced hNPC survival through increased expression of cIAP2, and mediated neuroprotection by regulating the M1/M2 phenotypes through increased expression of CX3CL1 and enhancing the expression of neurotrophic factors. Our results suggest some links between TNF-α-pretreated hNPC transplantation and neuroprotection and improved neurological functions after HI brain injury. Therefore, TNF-α pretreatment may be a simple and safe approach to protect implanted hNPCs against HI conditions and improve the therapeutic effectiveness of hNPC-based cell therapy in HI brain injury.

## Abbreviations

TNF-α	Tumor necrosis factor-α
hNPCs	Human neural progenitor cells
HI	Hypoxic-ischemic
cIAP2	cellular inhibitor of apoptosis 2
shRNA	short hairpin RNA
BLI	Bioluminescent imaging
ELISA	Enzyme-linked immunosorbent assay
FBS	Fetal bovine serum
DMEM/F12	Dulbecco’s modified Eagle’s medium and Ham’s F-12 medium
DMEM	Dulbecco’s modified Eagles medium
PBS	Phosphate-buffered saline
LIF	leukemia inhibitory factor
FGF2	fibroblast growth factor-2
CM	conditioned media
NSS	Neurological severity score
NDS	normal donkey serum
BSA	bovine serum albumin
Iba1	Ionized calcium-binding adaptor molecule
NGF	Nerve growth factor
NT3	Neurotrophin 3
NT4	Neurotrophin 4
VEGF	vascular endothelial growth factor
TUNEL	Terminal deoxynucleotidyl transferase-mediated dUTP nick end labeling
ELISA	Enzyme-linked immunosorbent assay
ANOVA	Analysis of variance
SEM	Standard error of the mean
BDNF	Brain-derived neurotrophic factor
p/s/cm2/sr	photons per second per square centimeter per steridian
LV LTR	lentivirus long term repeat
EF1-α	EF1-alpha promoter
WPRE	the post transcriptional regulatory element of woodchuck hepatitis virus
SIN LTR	self-inactivating lentiviral long terminal repeat

## References

[1] Volpe J.J. (2012). Neonatal encephalopathy: An inadequate term for hypoxic-ischemic encephalopathy. Ann. Neurol..

[2] Ferriero D.M. (2004). Neonatal brain injury. N. Engl. J. Med..

[3] Gluckman P.D., Wyatt J.S., Azzopardi D., Ballard R., Edwards A.D., Ferriero D.M., Polin R.A., Robertson C.M., Thoresen M., Whitelaw A. (2005). Selective head cooling with mild systemic hypothermia after neonatal encephalopathy: Multicentre randomised trial. Lancet.

[4] Darsalia V., Allison S.J., Cusulin C., Monni E., Kuzdas D., Kallur T., Lindvall O., Kokaia Z. (2011). Cell number and timing of transplantation determine survival of human neural stem cell grafts in stroke-damaged rat brain. J. Cereb. Blood Flow Metab..

[5] Bliss T.M., Andres R.H., Steinberg G.K. (2010). Optimizing the success of cell transplantation therapy for stroke. Neurobiol. Dis..

[6] Andres R.H., Horie N., Slikker W., Keren-Gill H., Zhan K., Sun G., Manley N.C., Pereira M.P., Sheikh L.A., McMillan E.L. (2011). Human neural stem cells enhance structural plasticity and axonal transport in the ischaemic brain. Brain.

[7] Hicks A.U., Lappalainen R.S., Narkilahti S., Suuronen R., Corbett D., Sivenius J., Hovatta O., Jolkkonen J. (2009). Transplantation of human embryonic stem cell-derived neural precursor cells and enriched environment after cortical stroke in rats: Cell survival and functional recovery. Eur. J. Neurosci..

[8] Nakagomi N., Nakagomi T., Kubo S., Nakano-Doi A., Saino O., Takata M., Yoshikawa H., Stern D.M., Matsuyama T., Taguchi A. (2009). Endothelial cells support survival, proliferation, and neuronal differentiation of transplanted adult ischemia-induced neural stem/progenitor cells after cerebral infarction. Stem Cells.

[9] Gage F.H., Temple S. (2013). Neural stem cells: Generating and regenerating the brain. Neuron.

[10] Lindvall O., Kokaia Z. (2010). Stem cells in human neurodegenerative disorders--time for clinical translation?. J. Clin. Investig..

[11] Goldman S.A. (2016). Stem and progenitor cell-based therapy of the central nervous system: Hopes, hype, and wishful thinking. Cell Stem Cell.

[12] Liu H., Honmou O., Harada K., Nakamura K., Houkin K., Hamada H., Kocsis J.D. (2006). Neuroprotection by plgf gene-modified human mesenchymal stem cells after cerebral ischaemia. Brain.

[13] Lee H.J., Kim M.K., Kim H.J., Kim S.U. (2009). Human neural stem cells genetically modified to overexpress akt1 provide neuroprotection and functional improvement in mouse stroke model. PLoS ONE.

[14] Lee I.S., Koo K.Y., Jung K., Kim M., Kim I.S., Hwang K., Yun S., Lee H., Shin J.E., Park K.I. (2017). Neurogenin-2-transduced human neural progenitor cells attenuate neonatal hypoxic-ischemic brain injury. Transl. Res..

[15] Sakata H., Niizuma K., Wakai T., Narasimhan P., Maier C.M., Chan P.H. (2012). Neural stem cells genetically modified to overexpress cu/zn-superoxide dismutase enhance amelioration of ischemic stroke in mice. Stroke.

[16] Wang F., Kameda M., Yasuhara T., Tajiri N., Kikuchi Y., Liang H.B., Tayra J.T., Shinko A., Wakamori T., Agari T. (2011). Gdnf-pretreatment enhances the survival of neural stem cells following transplantation in a rat model of parkinson’s disease. Neurosci. Res..

[17] Sakata H., Narasimhan P., Niizuma K., Maier C.M., Wakai T., Chan P.H. (2012). Interleukin 6-preconditioned neural stem cells reduce ischaemic injury in stroke mice. Brain.

[18] Rosenblum S., Smith T.N., Wang N., Chua J.Y., Westbroek E., Wang K., Guzman R. (2015). Bdnf pretreatment of human embryonic-derived neural stem cells improves cell survival and functional recovery after transplantation in hypoxic-ischemic stroke. Cell Transplant..

[19] Hallenbeck J.M. (2002). The many faces of tumor necrosis factor in stroke. Nat. Med..

[20] Schwabe R.F., Brenner D.A. (2006). Mechanisms of liver injury. I. Tnf-alpha-induced liver injury: Role of ikk, jnk, and ros pathways. Am. J. Physiol. Gastrointest. Liver Physiol..

[21] Bernardino L., Agasse F., Silva B., Ferreira R., Grade S., Malva J.O. (2008). Tumor necrosis factor-alpha modulates survival, proliferation, and neuronal differentiation in neonatal subventricular zone cell cultures. Stem Cells.

[22] Lan X., Chen Q., Wang Y., Jia B., Sun L., Zheng J., Peng H. (2012). Tnf-alpha affects human cortical neural progenitor cell differentiation through the autocrine secretion of leukemia inhibitory factor. PLoS ONE.

[23] Peng H., Whitney N., Wu Y., Tian C., Dou H., Zhou Y., Zheng J. (2008). Hiv-1-infected and/or immune-activated macrophage-secreted tnf-alpha affects human fetal cortical neural progenitor cell proliferation and differentiation. Glia.

[24] Kim M., Jung K., Kim I.S., Lee I.S., Ko Y., Shin J.E., Park K.I. (2018). Tnf-alpha induces human neural progenitor cell survival after oxygen-glucose deprivation by activating the nf-kappab pathway. Exp. Mol. Med..

[25] Kim H.T., Kim I.S., Lee I.S., Lee J.P., Snyder E.Y., Park K.I. (2006). Human neurospheres derived from the fetal central nervous system are regionally and temporally specified but are not committed. Exp. Neurol..

[26] Trapnell C., Pachter L., Salzberg S.L. (2009). Tophat: Discovering splice junctions with rna-seq. Bioinformatics.

[27] Langmead B., Salzberg S.L. (2012). Fast gapped-read alignment with bowtie 2. Nat. Methods.

[28] Trapnell C., Roberts A., Goff L., Pertea G., Kim D., Kelley D.R., Pimentel H., Salzberg S.L., Rinn J.L., Pachter L. (2012). Differential gene and transcript expression analysis of rna-seq experiments with tophat and cufflinks. Nat. Protoc..

[29] Leviton A., Dammann O., Durum S.K. (2005). The adaptive immune response in neonatal cerebral white matter damage. Ann. Neurol..

[30] Aden U., Favrais G., Plaisant F., Winerdal M., Felderhoff-Mueser U., Lampa J., Lelievre V., Gressens P. (2010). Systemic inflammation sensitizes the neonatal brain to excitotoxicity through a pro-/anti-inflammatory imbalance: Key role of tnfalpha pathway and protection by etanercept. Brain Behav. Immun..

[31] Lai A.Y., Todd K.G. (2006). Microglia in cerebral ischemia: Molecular actions and interactions. Can. J. Physiol. Pharmacol..

[32] Banati R.B., Gehrmann J., Schubert P., Kreutzberg G.W. (1993). Cytotoxicity of microglia. Glia.

[33] Hermann D.M., Peruzzotti-Jametti L., Schlechter J., Bernstock J.D., Doeppner T.R., Pluchino S. (2014). Neural precursor cells in the ischemic brain—Integration, cellular crosstalk, and consequences for stroke recovery. Front. Cell Neurosci..

[34] Sakata H., Niizuma K., Yoshioka H., Kim G.S., Jung J.E., Katsu M., Narasimhan P., Maier C.M., Nishiyama Y., Chan P.H. (2012). Minocycline-preconditioned neural stem cells enhance neuroprotection after ischemic stroke in rats. J. Neurosci..

[35] Liu F., Xuan A., Chen Y., Zhang J., Xu L., Yan Q., Long D. (2014). Combined effect of nerve growth factor and brainderived neurotrophic factor on neuronal differentiation of neural stem cells and the potential molecular mechanisms. Mol. Med. Rep..

[36] Turrin N.P., Rivest S. (2006). Tumor necrosis factor alpha but not interleukin 1 beta mediates neuroprotection in response to acute nitric oxide excitotoxicity. J. Neurosci..

[37] Polunovsky V.A., Wendt C.H., Ingbar D.H., Peterson M.S., Bitterman P.B. (1994). Induction of endothelial cell apoptosis by tnf alpha: Modulation by inhibitors of protein synthesis. Exp. Cell Res..

[38] Wang C.Y., Mayo M.W., Baldwin A.S. (1996). Tnf- and cancer therapy-induced apoptosis: Potentiation by inhibition of nf-kappab. Science.

[39] Pimentel-Coelho P.M., Magalhaes E.S., Lopes L.M., deAzevedo L.C., Santiago M.F., Mendez-Otero R. (2010). Human cord blood transplantation in a neonatal rat model of hypoxic-ischemic brain damage: Functional outcome related to neuroprotection in the striatum. Stem Cells Dev..

[40] Van Velthoven C.T., Kavelaars A., van Bel F., Heijnen C.J. (2010). Mesenchymal stem cell treatment after neonatal hypoxic-ischemic brain injury improves behavioral outcome and induces neuronal and oligodendrocyte regeneration. Brain Behav. Immun..

[41] Ye Q., Wu Y., Wu J., Zou S., Al-Zaazaai A.A., Zhang H., Shi H., Xie L., Liu Y., Xu K. (2018). Neural stem cells expressing bfgf reduce brain damage and restore sensorimotor function after neonatal hypoxia-ischemia. Cell Physiol. Biochem..

[42] Lee I.S., Jung K., Kim M., Park K.I. (2010). Neural stem cells: Properties and therapeutic potentials for hypoxic-ischemic brain injury in newborn infants. Pediatr. Int..

[43] Liston P., Roy N., Tamai K., Lefebvre C., Baird S., Cherton-Horvat G., Farahani R., McLean M., Ikeda J.E., MacKenzie A. (1996). Suppression of apoptosis in mammalian cells by naip and a related family of iap genes. Nature.

[44] Liston P., Young S.S., Mackenzie A.E., Korneluk R.G. (1997). Life and death decisions: The role of the iaps in modulating programmed cell death. Apoptosis.

[45] Tanaka H., Yokota H., Jover T., Cappuccio I., Calderone A., Simionescu M., Bennett M.V., Zukin R.S. (2004). Ischemic preconditioning: Neuronal survival in the face of caspase-3 activation. J. Neurosci..

[46] Conte D., Holcik M., Lefebvre C.A., Lacasse E., Picketts D.J., Wright K.E., Korneluk R.G. (2006). Inhibitor of apoptosis protein ciap2 is essential for lipopolysaccharide-induced macrophage survival. Mol. Cell. Biol..

[47] Dickson D.W., Lee S.C., Mattiace L.A., Yen S.H., Brosnan C. (1993). Microglia and cytokines in neurological disease, with special reference to aids and alzheimer’s disease. Glia.

[48] Chao C.C., Hu S., Peterson P.K. (1995). Glia, cytokines, and neurotoxicity. Crit. Rev. Neurobiol..

[49] Jin R., Yang G., Li G. (2010). Inflammatory mechanisms in ischemic stroke: Role of inflammatory cells. J. Leukoc. Biol..

[50] Hu X., Li P., Guo Y., Wang H., Leak R.K., Chen S., Gao Y., Chen J. (2012). Microglia/macrophage polarization dynamics reveal novel mechanism of injury expansion after focal cerebral ischemia. Stroke.

[51] Wang G., Zhang J., Hu X., Zhang L., Mao L., Jiang X., Liou A.K., Leak R.K., Gao Y., Chen J. (2013). Microglia/macrophage polarization dynamics in white matter after traumatic brain injury. J. Cereb. Blood Flow Metab..

[52] Prinz M., Priller J. (2014). Microglia and brain macrophages in the molecular age: From origin to neuropsychiatric disease. Nat. Rev. Neurosci..

[53] Cherry J.D., Olschowka J.A., O’Banion M.K. (2014). Neuroinflammation and m2 microglia: The good, the bad, and the inflamed. J. Neuroinflammation.

[54] Cruz-Guilloty F., Saeed A.M., Echegaray J.J., Duffort S., Ballmick A., Tan Y., Betancourt M., Viteri E., Ramkhellawan G.C., Ewald E. (2013). Infiltration of proinflammatory m1 macrophages into the outer retina precedes damage in a mouse model of age-related macular degeneration. Int. J. Inflam..

[55] Hellstrom Erkenstam N., Smith P.L., Fleiss B., Nair S., Svedin P., Wang W., Bostrom M., Gressens P., Hagberg H., Brown K.L. (2016). Temporal characterization of microglia/macrophage phenotypes in a mouse model of neonatal hypoxic-ischemic brain injury. Front. Cell Neurosci..

[56] Garcia G.E., Xia Y., Chen S., Wang Y., Ye R.D., Harrison J.K., Bacon K.B., Zerwes H.G., Feng L. (2000). Nf-kappab-dependent fractalkine induction in rat aortic endothelial cells stimulated by il-1beta, tnf-alpha, and lps. J. Leukoc. Biol..

[57] Fraticelli P., Sironi M., Bianchi G., D’Ambrosio D., Albanesi C., Stoppacciaro A., Chieppa M., Allavena P., Ruco L., Girolomoni G. (2001). Fractalkine (cx3cl1) as an amplification circuit of polarized th1 responses. J. Clin. Investig..

[58] Zujovic V., Benavides J., Vige X., Carter C., Taupin V. (2000). Fractalkine modulates tnf-alpha secretion and neurotoxicity induced by microglial activation. Glia.

[59] Wang J., Zhang X.S., Tao R., Zhang J., Liu L., Jiang Y.H., Ma S.H., Song L.X., Xia L.J. (2017). Upregulation of cx3cl1 mediated by nf-kappab activation in dorsal root ganglion contributes to peripheral sensitization and chronic pain induced by oxaliplatin administration. Mol. Pain.

[60] Mizuno T., Kawanokuchi J., Numata K., Suzumura A. (2003). Production and neuroprotective functions of fractalkine in the central nervous system. Brain Res..

[61] Sheikh A.M., Nagai A., Wakabayashi K., Narantuya D., Kobayashi S., Yamaguchi S., Kim S.U. (2011). Mesenchymal stem cell transplantation modulates neuroinflammation in focal cerebral ischemia: Contribution of fractalkine and il-5. Neurobiol. Dis..

[62] Wang Y., Fu Y., Xue S., Ai A., Chen H., Lyu Q., Kuang Y. (2014). The m2 polarization of macrophage induced by fractalkine in the endometriotic milieu enhances invasiveness of endometrial stromal cells. Int. J. Clin. Exp. Pathol..

[63] Castro-Sanchez S., Garcia-Yague A.J., Lopez-Royo T., Casarejos M., Lanciego J.L., Lastres-Becker I. (2018). Cx3cr1-deficiency exacerbates alpha-synuclein-A53T induced neuroinflammation and neurodegeneration in a mouse model of parkinson’s disease. Glia.

[64] Tang Z., Gan Y., Liu Q., Yin J.X., Liu Q., Shi J., Shi F.D. (2014). Cx3cr1 deficiency suppresses activation and neurotoxicity of microglia/macrophage in experimental ischemic stroke. J. Neuroinflammation.

[65] Zhang L., Xu J., Gao J., Wu Y., Yin M., Zhao W. (2018). Cd200-, cx3cl1-, and trem2-mediated neuron-microglia interactions and their involvements in Alzheimer’s disease. Rev. Neurosci..

[66] Febinger H.Y., Thomasy H.E., Pavlova M.N., Ringgold K.M., Barf P.R., George A.M., Grillo J.N., Bachstetter A.D., Garcia J.A., Cardona A.E. (2015). Time-dependent effects of cx3cr1 in a mouse model of mild traumatic brain injury. J. Neuroinflammation.

[67] Cardona A.E., Pioro E.P., Sasse M.E., Kostenko V., Cardona S.M., Dijkstra I.M., Huang D., Kidd G., Dombrowski S., Dutta R. (2006). Control of microglial neurotoxicity by the fractalkine receptor. Nat. Neurosci..

[68] Giunti D., Parodi B., Usai C., Vergani L., Casazza S., Bruzzone S., Mancardi G., Uccelli A. (2012). Mesenchymal stem cells shape microglia effector functions through the release of cx3cl1. Stem Cells.

[69] Luo P., Chu S.F., Zhang Z., Xia C.Y., Chen N.H. (2019). Fractalkine/CX3CR1 is involved in the cross-talk between neuron and glia in neurological diseases. Brain Res. Bull..

[70] Mehta A., Prabhakar M., Kumar P., Deshmukh R., Sharma P.L. (2013). Excitotoxicity: Bridge to various triggers in neurodegenerative disorders. Eur. J. Pharmacol..

[71] Holtzman D.M., Lee S., Li Y., Chua-Couzens J., Xia H., Bredt D.S., Mobley W.C. (1996). Expression of neuronal-nos in developing basal forebrain cholinergic neurons: Regulation by ngf. Neurochem. Res..

[72] Zhang A., Liang L., Niu H., Xu P., Hao Y. (2012). Protective effects of vegf treatment on focal cerebral ischemia in rats. Mol. Med. Rep..

[73] Zhang J., Shi Q., Yang P., Xu X., Chen X., Qi C., Zhang J., Lu H., Zhao B., Zheng P. (2012). Neuroprotection of neurotrophin-3 against focal cerebral ischemia/reperfusion injury is regulated by hypoxia-responsive element in rats. Neuroscience.

[74] Han B.H., Holtzman D.M. (2000). Bdnf protects the neonatal brain from hypoxic-ischemic injury in vivo via the erk pathway. J. Neurosci..

[75] Davies A.M. (1994). The role of neurotrophins in the developing nervous system. J. Neurobiol..

[76] Wang Y., Chang C.F., Morales M., Chiang Y.H., Hoffer J. (2002). Protective effects of glial cell line-derived neurotrophic factor in ischemic brain injury. Ann. N. Y. Acad. Sci..

[77] Fantacci C., Capozzi D., Ferrara P., Chiaretti A. (2013). Neuroprotective role of nerve growth factor in hypoxic-ischemic brain injury. Brain Sci..

